# Development of Danish version of child oral-health-related quality of life questionnaires (CPQ_8–10 _and CPQ_11–14_)

**DOI:** 10.1186/1472-6831-9-11

**Published:** 2009-04-22

**Authors:** Pia Wogelius, Hans Gjørup, Dorte Haubek, Rodrigo Lopez, Sven Poulsen

**Affiliations:** 1 Department of Paediatric Dentistry, School of Dentistry, Faculty of Health Sciences, Aarhus University, Vennelyst Boulevard 9, DK-8000 Aarhus, Denmark; 2 Department of Clinical Epidemiology, Aalborg Hospital Aarhus University Hospital, Søndre Skovvej 15, DK-9000 Aalborg, Denmark; 3 Resource Centre for Oral Health in Rare Medical Conditions, Aarhus University Hospital, Nørrebrogade 44, DK-8000 Aarhus, Denmark; 4 Department of Periodontology, School of Dentistry, Faculty of Health Sciences, Aarhus University, Vennelyst Boulevard 9, DK-8000 Aarhus, Denmark

## Abstract

**Background:**

The Child Perceptions Questionnaire (CPQ) is a self-reported questionnaire developed to measure oral health-related quality of life in children. The CPQ aims to improve the description of children's oral health, while taking into consideration the importance of psychological aspects in the concept of health. The CPQ exists in two versions: the CPQ_8–10 _for children aged 8–10 years and the CPQ_11–14 _for those aged 11–14 years. The aim of this study was to develop a Danish version of the CPQ_8–10 _and the CPQ_11–14 _and to evaluate its validity for use among Danish-speaking children.

**Methods:**

The instruments were translated from English into Danish in accordance with a recommended translation procedure. Afterwards, they were tested among children aged 8–10 (n = 120) and 11–14 years (n = 225). The validity was expressed by the correlation between overall CPQ scores and i) self-reported assessment of the influence of oral conditions on everyday life (not at all, very little, some, a lot, very much) and ii) the self-reported rating of oral health. Furthermore, groups of children with assumed decreased oral health-related quality of life were compared with children with healthy oral conditions. Finally, we examined the internal consistency.

**Results:**

The correlation between overall CPQ scores and global assessments of the influence of oral conditions on everyday life showed Spearman correlation coefficients of 0.45, *P < 0.001 *for CPQ_8–10 _and 0.50, *P < 0.001 *for CPQ_11–14_. The correlation between overall CPQ scores and the self-reported rating of oral health showed Spearman correlation coefficients of 0.45, *P < 0.001 *for CPQ_8–10 _and 0.17, P = 0.010 for CPQ_11–14_.

The median overall CPQ_8–10 _scores were 7 for individuals with healthy oral conditions, 5 for individuals with cleft lip and palate, and 15 for individuals with rare oral diseases. The median overall CPQ_11–14 _scores were 9 for individuals with healthy oral conditions, 9 for individuals with cleft lip and palate, 17.0 for individuals with rare oral diseases, and 22.0 for individuals with fixed orthodontic appliances. There were statistically significant differences between the groups of children with healthy oral conditions and each of the subgroups, except for children with cleft lip and palate.

Chronbach'α were 0.82 for CPQ_8–10 _and 0.87 for CPQ_11–14_.

**Conclusion:**

The results of this study reveal that the Danish CPQ_8–10 _and CPQ_11–14_, seem to be valid instruments for measuring oral health-related quality of life in children although its ability to discriminate between children with cleft lip and palate and healthy children seem to be limited.

## Background

Oral health-related quality of life (OHRQoL) is an aspect of dental health addressing the patient's self-perceived perception of whether his or her current oral health status has an impact upon his or her actual quality of life [1-4]. The OHRQoL may be measured by means of different written questionnaires, which take into account a number of relevant domains, *i.e*. pain, functional symptoms or social disability [3].

Measuring OHRQoL in children by means of questionnaires is associated with several challenges because the children's abilities to read, think in abstract terms and their age-related ability to understand the concepts used in the questionnaire should be taken into account [5,6]. Bearing in mind these challenges, the Child Perceptions Questionnaire (CPQ) was developed to measure the OHRQoL among children between the ages of 8 and 10 years (CPQ_8–10_) and between the ages of 11 and 14 years (CPQ_11–14_) [7,8]. The CPQ includes four domain subscales of oral symptoms, functional limitations, emotional well-being and social well-being.

The CPQ_8–10 _and CPQ_11–14 _have been shown to enjoy sufficient convergent validity, i.e. the self-reported health status correlates with the overall CPQ scores [7,8]. Three studies [8-10] demonstrated that the CPQ_11–14 _could be used to discriminate between groups with known different physical dental health status, but another study challenged this finding [11]. The CPQ_8–10 _seemed to have less discriminatory power than the CPQ_11–14 _[7]. The internal consistency of the CPQ_8–10 _and the CPQ_11–14 _has been estimated as sufficient, with Chronbach's alpha ranging from 0.81 to 0.91 [7,9,12].

The aim of this study was to develop a Danish version of the CPQ_8–10 _and the CPQ_11–14 _(Additional files 1 and 2) and to evaluate their convergent and discriminative validities and internal consistencies for use among Danish-speaking children.

## Methods

### Translation of the questionnaires

The English CPQ versions were translated into Danish using the forward-backward technique recommended by Behling and Law [13,14]. The translation from English into Danish was performed by two of the native-speaking Danish investigators (PW and HG). The first Danish version was back-translated into English by a bilingual dental hygienist with English as her first language. This translation was compared with the original questionnaires, which called for minor adjustments of the Danish version. Further adjustments of the wording were made after a pilot-test of the questionnaires in a class of a grade 4 children (N = 23). Finally, colleagues from the Department of Paediatric Dentistry, Karolinska Institutet, Stockholm, Sweden, who had encountered similar translation problems were consulted before final decisions were made.

### Final questionnaires

The Danish CPQ_8–10 _contains a total of 27 items: 2 global questions about dental health, 5 questions on oral symptoms, 5 questions on functional limitations, 5 questions on emotional well-being and 10 questions on social well-being.

The Danish CPQ_11–14 _contains a total of 39 questions: 2 global questions about dental health, 6 questions on oral symptoms, 9 questions on functional limitations, 9 questions on emotional well-being and 13 questions on social well-being.

The response format for all the questions is a Likert-like scale. Response options and scores were: "never" (scoring 0), "once or twice" (1), "sometimes" (2), "often" (3) and "every day or almost every day" (4).

For logistical reasons, we were not able to obtain data to assess the test-retest reliability.

### Study population

The CPQ_8–10 _questionnaire was answered by three groups of respondents: grade 4 pupils from a public school (healthy children), children with cleft lip and palate referred to and under treatment at the Cleft Lip and Palate Institute of Aarhus, and children with rare conditions like *amelogenesis imperfecta*, *hypohidrotic ectodermal dysplasia *or *multiple dental agenesis *referred to the Resource Centre for Oral Health in Rare Medical Conditions at Aarhus University Hospital.

The CPQ_11–14 _questionnaire was answered by four groups of respondents: grade 6 pupils from a public school (healthy children), children with cleft lip and palate referred to and under treatment at the Cleft Lip and Palate Institute of Aarhus, children with rare oral diseases referred to the Resource Centre for Oral Health in Rare Medical Conditions and children undergoing orthodontic treatment with fixed orthodontic appliances inserted within the last 3 months before answering the questionnaires.

### Statistics

#### Calculation of CPQ scores

Questionnaires with missing answers for more than 2 items were excluded from further analysis. In the remaining questionnaires, item answers with missing values were recoded as 0 (zero). For each individual, the domain scores and the total CPQ score were calculated by adding the item scores.

#### Construct validity

The correlations between the overall CPQ scores and i) the self-reported assessment of the influence of oral conditions on everyday life, and ii) the self-reported rating of the oral health were examined. The Spearman rank correlation coefficient was used to test the hypothesis that individuals reporting a negative influence of oral conditions on everyday life and poor oral health have higher CPQ scores than individuals reporting a positive influence or good oral health. The median CPQ scores were compared between healthy children and each of the 2 CPQ_8–10 _groups and each of the 3 CPQ_11–14 _groups. The Mann Whitney test was used to test the hypothesis that healthy children have lower median CPQ scores than individuals from the cleft lip and palate group, the group of children with rare oral diseases and the orthodontic group for CPQ_11–14_. Previous studies examining the discriminative validity of the CPQ questionnaires have reported mean CPQ scores [7-11]. We calculated mean CPQ scores to allow comparison with the scores reported in extant literature.

#### Internal consistency

The internal consistency was assessed for the total CPQ score and for each of the domain scores by means of Cronbach's reliability coefficient α and inter-item correlation coefficients [15].

All analyses were performed using the STATA version 10.0; STATA, Texas, USA.

## Results

A total of 123 children answered the CPQ_8–10 _and 236 answered the CPQ_11–14 _questionnaire. Questionnaires with more than 2 missing item answers were excluded from the analyses; thus, the final study populations consisted of 120 8-to-10-year-old children and 225 11-to-14-year-old children (Table 1).

**Table 1:** Number of children included in the study

	CPQ_8–10_	CPQ_11–14_
	
Group of children	Number of children	
Healthy children	97	154
Children with cleft lip and palate	15	21
Children with rare oral diseases	8	20
Children with fixed orthodontic appliances	-	30

Total	120	225

### CPQ 8–10

The self-reported assessments of the influence of oral conditions on everyday life and ratings of oral health were positively correlated with the overall CPQ_8–10 _scores and the domain scores (Table 2). The Spearman correlation coefficients ranged from 0.29 for oral symptoms to 0.45 for the overall CPQ_8–10 _score.

**Table 2:** Mean overall scores (with S.D.) and Spearman rank correlation coefficients between CPQ_8–10 _scores, and self-perceived ratings of the influence of the oral conditions on daily life and self-perceived ratings of oral health

	"How much do your teeth or mouth bother you in your everyday life?"					
			
	Not at all	A little Bit	Some	A lot	Spearman correlation coefficient	P-value
	n = 51	n = 60	n = 5	n = 4		
Overall score	6.18 (6.03)	9.87 (5.95)	15.20 (5.07)	22.0 (9.13)	0.45	<0.001
Oral symptoms	3.80 (2.82)	5.03 (2.64)	6.40 (3.51)	7.25 (3.50)	0.29	0.001
Functional limitations	1.10 (1.95)	2.07 (2.32)	2.60 (1.14)	7.25 (2.63)	0.39	<0.001
Emotional well-being	0.65 (1.25)	1.52 (1.58)	3.80 (2.17)	4.25 (3.86)	0.42	<0.001
Social well-being	0.63 (1.52)	1.25 (1.56)	2.40 (2.07)	3.25 (0.96)	0.43	<0.001

	"Would you say that the health of your teeth is.."					
			
	Very good	Good	O.K.	Poor		
			
	n = 25	n = 49	n = 40	n = 6		

Overall score	5.16 (5.54)	7.73 (6.18)	11.42 (6.00)	17.67 (9.75)	0.45	<0.001
Oral symptoms	3.32 (2.95)	4.06 (2.38)	5.83 (2.85)	7.00 (2.76)	0.38	<0.001
Functional limitations	0.76 (1.23)	1.67 (2.26)	2.30 (2.50)	4.83 (3.66)	0.34	<0.001
Emotional well-being	0.4 (0.87)	1.08 (1.51)	1.80 (1.86)	4.17 (2.64)	0.42	<0.001
Social well-being	0.68 (1.73)	0.92 (1.64)	1.50 (1.54)	1.67 (1.67)	0.35	<0.001

**Figure 1:**
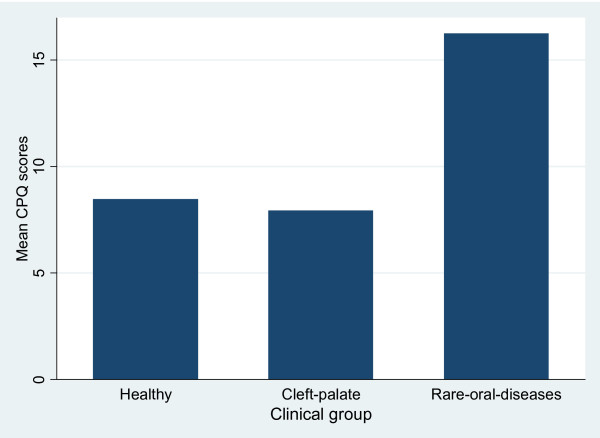
**Mean overall CPQ_8–10 _scores in healthy children, children with clef lip and palate, and in children with rare oral diseases**.

The highest median CPQ_8–10 _scores and domain scores were found among children with rare oral diseases, followed by healthy children, and children with cleft lip and palate (Table 3). There were statistically significant differences between healthy children and children with rare oral diseases, but not between healthy children and children with cleft lip and palate. The mean CPQ_8–10 _scores were 8.5 (SD: 6.2) among healthy children, 7.9 (SD: 8.0) among children with cleft lip and palate, and 16.3 (SD: 8.4) among children with rare oral diseases (Figure 1).

**Table 3:** Percentiles of overall CPQ_8–10 _score in 3 groups and CPQ_11–14 _score in 4 groups of children

CPQ_8–10_	CPQ_11–14_	Group of children			
		
		Healthy children	Children with cleft lip and palate	Children with rare oral diseases	Children with fixed orthodontic appliances
		
CPQ_8–10_	Quartiles				
	25 percentile	3	3	10	
	50 percentile	7	5	15	
	75 percentile	13	10	23.7	
	P-value	-	0.504	0.009	
		
CPQ_11–14_	Quartiles				
	25 percentile	5	5	9	16
	50 percentile	9	9	17	22
	75 percentile	13	17	26	32
	P-value	-	0.84	<0.001	<0.001

The P-values test whether the median overall CPQ-scores differ between the healthy children and each of the other subgroups.

Chronbach's α values values ranged from 0.57 for oral symptoms to 0.82 for the overall CPQ_8–10 _score (Table 4).

**Table 4:** The internal consistency and inter-item correlation for CPQ_8–10 _and CPQ_11–14 _and their subdomains

	Chronbach's α		Average inter-item correlations	
	
	CPQ_8–10_	CPQ_11–14_	CPQ_8–10_	CPQ_11–14_
Overall score	0.82	0.87	0.15	0.16
Oral symptoms	0.57	0.49	0.21	0.14
Functional limitations	0.78	0.69	0.37	0.20
Emotional well-being	0.76	0.85	0.34	0.40
Social well-being	0.69	0.68	0.17	0.14

### CPQ 11–14

The self-reported assessment of the influence of oral conditions on every day life correlated with the overall CPQ_11–14 _scores and each of the domain scores (Table 5). The Spearman correlation coefficients ranged from 0.30 for oral symptoms to 0.50 for the overall CPQ_11–14 _score. The self-reported ratings of oral health correlated with the overall CPQ_11–14 _scores, but not with the domain scores on functional limitations, emotional well-being or social well-being. Furthermore, the CPQ scores did not increase with decreasing self-reported oral health from the answer category "Excellent" to the answer category "Very good".

**Table 5:** Mean overall CPQ_11–14 _scores (with S.D.) and Spearman rank correlation coefficients between CPQ scores, and self-perceived ratings of the influence of oral conditions on daily life and self-perceived ratings of oral health

	"How much do your teeth or mouth bother you in your everyday life?"						
			
	Not at all	Very little	Some	A lot	Very much	Spearman correlation coefficient	P-value
	n = 56	n = 94	n = 36	n = 25	n = 11		
Overall score	5.96 (3.98)	13.20 (9.15)	16.97 (9.61)	19.28 (12.01)	18.27 (10.74)	0.50	<0.001
Oral symptoms	3.39 (2.02)	4.80 (2.57)	5.67 (2.91)	5.72 (2.62)	4.73 (3.88)	0.30	<0.001
Functional limitations	1.11 (1.72)	2.79 (2.79)	3.86 (3.48)	4.88 (5.25)	5.00 (4.40)	0.38	<0.001
Emotional well-being	0.75 (1.40)	3.54 (4.36)	4.53 (3.68)	5.92 (4.58)	5.91 (6.44)	0.46	<0.001
Social well-being	0.71 (1.00)	2.07 (2.42)	2.91 (2.70)	2.76 (3.19)	2.63 (2.58)	0.32	<0.001

	"Would you say that the health of your teeth is.."						
			
	Excellent	Very good	Good	Fair	Poor		
			
	n = 7	n = 82	n = 111	n = 20	n = 2		

Overall score	12.28 (10.90)	11.26 (8.09)	13.42 (10.80)	16.40 (9.59)	19.50 (0.71)	0.17	0.010
Oral symptoms	4.71 (2.93)	4.28 (2.77)	4.86 (2.70)	5.05 (2.19)	7.5 (2.12)	0.16	0.017
Functional limitations	3.29 (3.82)	2.34 (2.85)	3.09 (3.82)	3.65 (2.64)	3.50 (3.54)	0.14	0.038
Emotional well-being	2.86 (2.91)	2.79 (3.15)	3.46 (4.73)	5.35 (5.22)	5.00 (1.41)	0.09	0.167
Social well-being	1.43 (2.15)	1.84 (1.95)	2.01 (2.83)	2.35 (1.95)	3.50 (2.12)	0.08	0.256

**Figure 2:**
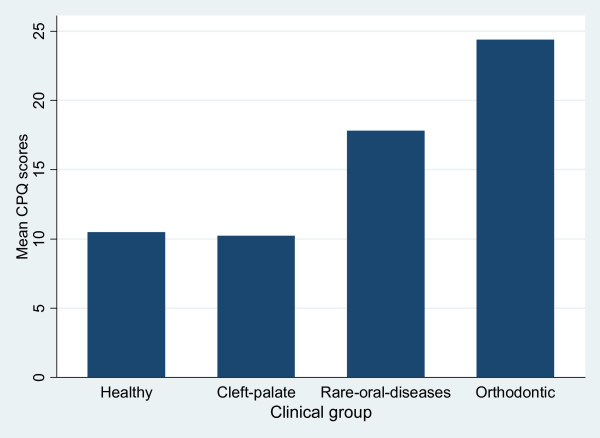
**Mean overall CPQ_11–14 _scores in healthy children, children with clef lip and palate, children with rare oral diseases, and in children with fixed orthodontic appliances**.

The highest CPQ_11–14 _scores and domain scores were found among children with orthodontic appliances placed within the last 3 months before answering the questionnaires (Table 3), followed by children with rare oral diseases, children with cleft lip and palate and healthy children. The median test showed statistically significant differences between the medians of the CPQ_11–14 _scores (P < 0.001). The mean CPQ_11–14 _scores were 10.5 (SD: 7.6) for healthy children, 10.2 (SD: 7.2) for children with cleft lip and palate, 17.8 (SD: 8.8) for children with rare oral diseases and 24.4 (SD: 12.5) among children with orthodontic appliances (Figure 2).

Chronbach's α values ranged from 0.49 for oral symptoms to 0.87 for the overall CPQ_11–14 _score (Table 4).

## Discussion

The study examined the validity of two versions of the CPQ_8–10 _and CPQ_11–14_, and the instruments seem to have appropriate construct validity and internal consistency among Danish-speaking children between the ages of 8 and 14 years.

The translation procedure from English into Danish raised several issues for consideration. In the original, English wording of the questions, only the first question on each page contained the full wording of the question (viz. "How often have you ....."). After the first question, the next questions were shortened versions, i.e. the opening phrase "How often have you" was left out. In the Danish translation, the opening line was repeated in all questions to exclude any doubt about the wording of the questions, even if the addition of the wording raised some concern about the length of the questions. A number of questions caused problems during the translation process, for example "How often have you had a hard time biting or chewing food like apples, corn on the cob...", because "corn on the cob" only recently became a widespread dish in Denmark. Although some of the questions contained built-in problems like in the above example, we found that all of the questions could be used in the Danish context [14,16]. In addition, Swedish colleagues engaged in similar translation problems were consulted before deciding on the final versions. Furthermore, we considered the domains of oral-health-related-quality of life identified by Jokovic et al. as well ad their names to be relevant and understandable for Danish children.

Children with more than 2 missing items were excluded from further analysis. There were only few instances of non-response and no specific pattern of non-response could be ascertained. The effect of the absence of responses was therefore considered to be minor.

Like the majority of Danish children, all of the included children were pupils of public schools and therefore most likely representative of the Danish child population.

The study showed that the overall CPQ scores correlated well with the global assessment of the influence of dental health on everyday life. This is in agreement with previous studies on the CPQ questionnaire [7,8]. However, for the CPQ_8–10_, the correlation between the global assessment of the influence of the dental health on everyday life and the CPQ scores was low for the symptom scores and high for the scores on social well-being. This contrasted with the findings by Jokovic et al [7]. One possible explanation is that 8-to-10-year-olds are familiar with oral symptoms, among others due to loose primary teeth, and these symptoms may have less impact on their everyday life than similar symptoms in the older group. In the daily clinical situation, dental health carers will, most likely, be concerned about oral symptoms when they evaluate the patient's oral health, but, as demonstrated, this is not necessarily the most important component of the OHRQoL among the 8-to-10-year-olds. This finding underlines the value of considering broader aspects of the dental health in children than only the physical ones.

Three of the CPQ_11–14 _domain scores did not correlate with the self-reported assessment of oral health. A possible explanation is that the 11–14 year-olds consider their teeth to be healthy if caries-free, while the CPQ_11–14 _questions explore emotional and social aspects which may dominate in the minds of the 11–14-year-olds. Furthermore, the CPQ scores did not increase with decreasing self-reported oral health from the answer category "Excellent" to the answer category "Very good", which may simply be so because it can be semantically difficult to distinguish between "Very good" and "Excellent".

In agreement with the majority of previous studies, the CPQ allowed us to discriminate between groups with known differences in dental health [8-10]. A remarkable, high CPQ_11–14 _score was observed among children with fixed orthodontic appliances and, in contrast to another study [8], the CPQ scores of orthodontic patients exceeded those of the cleft lip and palate patients. We may have obtained this result because our inclusion criteria restricted orthodontic patients to subjects with newly inserted fixed appliances. As shown in previous studies, pain and discomfort are most pronounced during the first period after insertion of the appliances [17-19]. Furthermore, Sergl et al. showed that patients with fixed appliances experience more discomfort than patients with removable appliances [17]. Surprisingly, children with cleft lip and palate reported CPQ-scores similar to those reported by healthy children. This finding disagreed with previous studies comparing CPQ-scores among children with cleft lip and palate and healthy children [8] and the observation may question the discriminative validity of the instrument. Another possible explanation is that cleft lip and palate is a chronic disorder which allows the children time to adapt to their situation.

Even though quality of life is a subjective perception, parents are frequently used as informants on children's health. However, previous studies have shown less than optimal agreement between parents' and children's rating of OHRQoL [20]. It is therefore essential to be able to measure self-reported OHRQoL in children.

Larger, population-based studies on representative groups of children are needed to establish normative data on oral-health- related quality of life and its determinants in Danish children.

## Conclusion

The results of this study reveal that the Danish CPQ_8–10 _and CPQ_11–14_, seem to be valid instruments for measuring oral health-related quality of life in children although its ability to discriminate between children with cleft lip and palate and healthy children seem to be limited.

## Abbreviations

OHRQoL: Oral health-related quality of life; CPQ: Child Perceptions Questionnaire

## Competing interests

The authors declare that they have no competing interests.

## Authors' contributions

PW and HG translated and developed the Danish version of the CPQ. PW, DH, HG and SP designed the study. PW, HG and DH and collected the data. PW and RL analyzed the data. PW drafted the manuscript and RL, DH, HG and SP reviewed, edited and approved the manuscript.

## Pre-publication history

The pre-publication history for this paper can be accessed here:



## Supplementary Material

Additional file 1 **The Danish version of the CPQ_8–10_**. The questionnaire is the Danish version of the Child Perceptions Questionnaire (CPQ) which measures the Oral health-related quality of life among children between the ages of 8 and 10 years (CPQ_8–10_). Click here for file

Additional file 2 **The Danish version of the CPQ_11–14_**. The questionnaire is the Danish version of the Child Perceptions Questionnaire (CPQ) which measures the Oral health-related quality of life among children between the ages of 11 and 14 years (CPQ_11–14_). Click here for file
