# Supplementary material for: Development of Danish version of child oral-health-related quality of life questionnaires (CPQ8–10 and CPQ11–14)
Source: BMC Oral Health. 2009 Apr 22;9:11. doi: 10.1186/1472-6831-9-11 (PMC2679003; doi:10.1186/1472-6831-9-11)
Supplement: Additional file 1 — The Danish version of the CPQ8–10. The questionnaire is the Danish version of the Child Perceptions Questionnaire (CPQ) which measures the Oral health-related quality of life among children between the ages of 8 and 10 years (CPQ8–10). [file 1472-6831-9-11-S1.doc]

**SPØRGESKEMA OM TÆNDER OG TRIVSEL**

Datoen i dag: _______/__________/________

dag måned år

1. Er du en dreng eller en pige?

⁪ Dreng

⁪ Pige

**2.** Hvornår blev du født? _______/__________/________ Alder__________

dag måned år

1. Når du tænker på dine tænder eller din mund, vil du så sige de er:

⁪ Meget gode

⁪ Gode

⁪ I orden

⁪ Dårlige

1. Hvor meget generer dine tænder eller din mund dig til hverdag?

⁪ Slet ikke

⁪ En lille smule

⁪ Noget

⁪ Meget

**Nu nogle få spørgsmål om dine tænder og din mund**

1. Hvor ofte har du i de sidste 4 uger haft **ondt i dine tænder eller din mund**?

⁪ Aldrig

⁪ En eller to gange

⁪ Sommetider

⁪ Tit

⁪ Hver dag, eller næsten hver dag

1. Hvor ofte har du i de sidste 4 uger haft **sår i munden**?

⁪ Aldrig

⁪ En eller to gange

⁪ Sommetider

⁪ Tit

⁪ Hver dag, eller næsten hver dag

1. Hvor ofte har du i de sidste 4 uger haft **ondt i tænderne, når du drikker kolde drikke eller spiser varm mad**?

⁪ Aldrig

⁪ En eller to gange

⁪ Sommetider

⁪ Tit

⁪ Hver dag, eller næsten hver dag

1. Hvor ofte har du i de sidste 4 uger haft **mad der sad fast i dine tænder**?

⁪ Aldrig

⁪ En eller to gange

⁪ Sommetider

⁪ Tit

⁪ Hver dag, eller næsten hver dag

1. Hvor ofte har du i de sidste 4 uger haft **dårlig ånde**?

⁪ Aldrig

⁪ En eller to gange

⁪ Sommetider

⁪ Tit

⁪ Hver dag, eller næsten hver dag

1. Hvor ofte har du i de sidste 4 uger på grund af dine tænder eller din mund **haft brug for mere tid end andre til at spise dine måltider**?

⁪ Aldrig

⁪ En eller to gange

⁪ Sommetider

⁪ Tit

⁪ Hver dag, eller næsten hver dag

1. Hvor ofte har du i de sidste 4 uger på grund af dine tænder eller din mund **haft svært ved at bide eller tygge æbler, majskolber eller kød?**

⁪ Aldrig

⁪ En eller to gange

⁪ Sommetider

⁪ Tit

⁪ Hver dag, eller næsten hver dag

1. Hvor ofte har du i de sidste 4 uger på grund af dine tænder eller din mund **haft svært ved at spise noget, du godt kan lide?**

⁪ Aldrig

⁪ En eller to gange

⁪ Sommetider

⁪ Tit

⁪ Hver dag, eller næsten hver dag

1. Hvor ofte har du i de sidste 4 uger **haft svært ved at sige nogle ord** på grund af dine tænder eller din mund i?

⁪ Aldrig

⁪ En eller to gange

⁪ Sommetider

⁪ Tit

⁪ Hver dag, eller næsten hver dag

1. Hvor ofte har du i de sidste 4 uger **haft problemer med at sove om natten** på grund af dine tænder eller din mund?

⁪ Aldrig

⁪ En eller to gange

⁪ Sommetider

⁪ Tit

⁪ Hver dag, eller næsten hver dag

**Nogle spørgsmål om dine følelser**

1. Hvor ofte har du i de sidste 4 uger **været ked af det eller irriteret** på grund af dine tænder eller din mund?

⁪ Aldrig

⁪ En eller to gange

⁪ Sommetider

⁪ Tit

⁪ Hver dag, eller næsten hver dag

1. Hvor ofte har du i de sidste 4 uger **følt dig skuffet eller modløs** på grund af dine tænder eller din mund?

⁪ Aldrig

⁪ En eller to gange

⁪ Sommetider

⁪ Tit

⁪ Hver dag, eller næsten hver dag

1. Hvor ofte har du i de sidste 4 uger **været genert** på grund af dine tænder eller din mund?

⁪ Aldrig

⁪ En eller to gange

⁪ Sommetider

⁪ Tit

⁪ Hver dag, eller næsten hver dag

1. Hvor ofte har du i de sidste 4 uger **været bekymret over hvad andre mennesker tænker om dine tænder eller din mund**?

⁪ Aldrig

⁪ En eller to gange

⁪ Sommetider

⁪ Tit

⁪ Hver dag, eller næsten hver dag

1. Hvor ofte har du i de sidste 4 uger på grund af dine tænder eller din mund **været bekymret over, om du ser lige så godt ud som andre?**

⁪ Aldrig

⁪ En eller to gange

⁪ Sommetider

⁪ Tit

⁪ Hver dag, eller næsten hver dag

S**pørgsmål om din skole**

1. Hvor ofte har du i de sidste 4 uger **været væk fra skolen** på grund af dine tænder eller din mund?

⁪ Aldrig

⁪ En eller to gange

⁪ Sommetider

⁪ Tit

⁪ Hver dag, eller næsten hver dag

1. Hvor ofte har du i de sidste 4 uger **haft svært ved at lave lektier** på grund af dine tænder eller din mund?

⁪ Aldrig

⁪ En eller to gange

⁪ Sommetider

⁪ Tit

⁪ Hver dag, eller næsten hver dag

1. Hvor ofte har du i de sidste 4 uger **haft svært ved at være opmærksom i skolen** på grund af dine tænder eller din mund?

⁪ Aldrig

⁪ En eller to gange

⁪ Sommetider

⁪ Tit

⁪ Hver dag, eller næsten hver dag

1. Hvor ofte har du i de sidste 4 uger **ikke ønsket at tale eller læse højt i klassen** på grund af dine tænder eller din mund?

⁪ Aldrig

⁪ En eller to gange

⁪ Sommetider

⁪ Tit

⁪ Hver dag, eller næsten hver dag

S**pørgsmål om dit samvær med andre mennesker**

1. Hvor ofte har du i de sidste 4 uger på grund af dine tænder eller din mund **forsøgt at lade være med at smile eller le, når du er sammen med andre børn** ?

⁪ Aldrig

⁪ En eller to gange

⁪ Sommetider

⁪ Tit

⁪ Hver dag, eller næsten hver dag

1. Hvor ofte har du i de sidste 4 uger **ikke haft lyst til at snakke med andre børn** på grund af dine tænder eller din mund?

⁪ Aldrig

⁪ En eller to gange

⁪ Sommetider

⁪ Tit

⁪ Hver dag, eller næsten hver dag

1. Hvor ofte har du i de sidste 4 uger **ikke haft lyst til at være sammen med andre børn** på grund af dine tænder eller din mund?

⁪ Aldrig

⁪ En eller to gange

⁪ Sommetider

⁪ Tit

⁪ Hver dag, eller næsten hver dag

1. Hvor ofte har du i de sidste 4 uger **holdt dig væk fra aktiviteter som sport eller klub** på grund af dine tænder eller din mund?

⁪ Aldrig

⁪ En eller to gange

⁪ Sommetider

⁪ Tit

⁪ Hver dag, eller næsten hver dag

1. Hvor ofte i de sidste 4 uger har **andre børn drillet dig eller givet dig øgenavne** på grund af dine tænder eller din mund?

⁪ Aldrig

⁪ En eller to gange

⁪ Sommetider

⁪ Tit

⁪ Hver dag, eller næsten hver dag

1. Hvor ofte i de sidste 4 uger har **andre børn stillet dig spørgsmål** om dine tænder eller din mund?

⁪ Aldrig

⁪ En eller to gange

⁪ Sommetider

⁪ Tit

⁪ Hver dag, eller næsten hver dag

**Mange tak for din hjælp!**
