# Supplementary material for: Development of Danish version of child oral-health-related quality of life questionnaires (CPQ8–10 and CPQ11–14)
Source: BMC Oral Health. 2009 Apr 22;9:11. doi: 10.1186/1472-6831-9-11 (PMC2679003; doi:10.1186/1472-6831-9-11)
Supplement: Additional file 2 — The Danish version of the CPQ11–14. The questionnaire is the Danish version of the Child Perceptions Questionnaire (CPQ) which measures the Oral health-related quality of life among children between the ages of 11 and 14 years (CPQ11–14). [file 1472-6831-9-11-S2.doc]

**TÆNDER OG TRIVSEL**

Datoen i dag: _______/__________/________

dag måned år

**Først nogle få spørgsmål om dig**

1. Er du dreng eller pige?

⁪ Dreng

⁪ Pige

1. Hvornår er du født: __________/________/___________

dag måned år

1. Vil du sige at dine tænders, munds, læbers og kæbers sundhed er:

⁪ Fortræffelig

⁪ Meget god

⁪ God

⁪ Nogenlunde

⁪ Dårlig

1. Hvor meget påvirker dine tænders, munds, læbers og kæbers tilstand dit liv som helhed?

⁪ Slet ikke

⁪ En lille smule

⁪ Noget

⁪ Meget

⁪ Rigtigt meget

**Spørgsmål om problemer omkring din mund**

1. Hvor ofte har du haft **ondt i dine tænder, mund, læber eller kæber** i de sidste 3 måneder?

⁪ Aldrig

⁪ En eller to gange

⁪ Sommetider

⁪ Tit

⁪ Hver dag, eller næsten hver dag

1. Hvor ofte har du haft **blødende tandkød** i de sidste 3 måneder?

⁪ Aldrig

⁪ En eller to gange

⁪ Sommetider

⁪ Tit

⁪ Hver dag, eller næsten hver dag

1. Hvor ofte har du haft **sår i munden** i de sidste 3 måneder?

⁪ Aldrig

⁪ En eller to gange

⁪ Sommetider

⁪ Tit

⁪ Hver dag, eller næsten hver dag

1. Hvor ofte har du haft **dårlig ånde** i de sidste 3 måneder?

⁪ Aldrig

⁪ En eller to gange

⁪ Sommetider

⁪ Tit

⁪ Hver dag, eller næsten hver dag

1. Hvor ofte har du haft **mad til at hænge fast i/mellem tænderne** i de sidste 3 måneder?

⁪ Aldrig

⁪ En eller to gange

⁪ Sommetider

⁪ Tit

⁪ Hver dag, eller næsten hver dag

1. Hvor ofte har du haft **mad til at sidde fast oppe i ganen** i de sidste 3 måneder?

⁪ Aldrig

⁪ En eller to gange

⁪ Sommetider

⁪ Tit

⁪ Hver dag, eller næsten hver dag

1. Hvor ofte i de sidste 3 måneder har du **oplevet at du kun kunne få vejret gennem munden** på grund af dine tænder, læber, mund eller kæbe?

⁪ Aldrig

⁪ En eller to gange

⁪ Sommetider

⁪ Tit

⁪ Hver dag, eller næsten hver dag

1. Hvor ofte i de sidste 3 måneder har du **brugt længere tid end andre til at spise et måltid** på grund af dine tænder, læber, mund eller kæbe?

⁪ Aldrig

⁪ En eller to gange

⁪ Sommetider

⁪ Tit

⁪ Hver dag, eller næsten hver dag

1. Hvor ofte i de sidste 3 måneder har du **haft søvnproblemer** på grund af dine tænder, læber, mund eller kæbe?

⁪ Aldrig

⁪ En eller to gange

⁪ Sommetider

⁪ Tit

⁪ Hver dag, eller næsten hver dag

1. Hvor ofte i de sidste 3 måneder har det **været svært for dig at bide eller tygge mad som æbler, majskolber eller kød** på grund af dine tænder, læber, mund eller kæbe?

⁪ Aldrig

⁪ En eller to gange

⁪ Sommetider

⁪ Tit

⁪ Hver dag, eller næsten hver dag

1. Hvor ofte i de sidste 3 måneder har det **været svært for dig at gabe højt** på grund af dine tænder, læber, mund eller kæbe?

⁪ Aldrig

⁪ En eller to gange

⁪ Sommetider

⁪ Tit

⁪ Hver dag, eller næsten hver dag

1. Hvor ofte i de sidste 3 måneder har det **været svært for dig at udtale nogle ord** på grund af dine tænder, læber, mund eller kæbe?

⁪ Aldrig

⁪ En eller to gange

⁪ Sommetider

⁪ Tit

⁪ Hver dag, eller næsten hver dag

1. Hvor ofte i de sidste 3 måneder har det på grund af dine tænder, læber, mund eller kæbe **været svært for dig at spise mad som du gerne vil have**?

⁪ Aldrig

⁪ En eller to gange

⁪ Sommetider

⁪ Tit

⁪ Hver dag, eller næsten hver dag

1. Hvor ofte i de sidste 3 måneder har det **været svært for dig at drikke med sugerør** på grund af dine tænder, læber, mund eller kæbe?

⁪ Aldrig

⁪ En eller to gange

⁪ Sommetider

⁪ Tit

⁪ Hver dag, eller næsten hver dag

1. Hvor ofte i de sidste 3 måneder har det **været svært for dig at drikke/spise koldt eller varmt** på grund af dine tænder, læber, mund eller kæbe?

⁪ Aldrig

⁪ En eller to gange

⁪ Sommetider

⁪ Tit

⁪ Hver dag, eller næsten hver dag

**Spørgsmål om følelser**

1. Hvor ofte i de sidste 3 måneder har du **følt dig irritabel eller skuffet** på grund af dine tænder, læber, mund eller kæbe?

⁪ Aldrig

⁪ En eller to gange

⁪ Sommetider

⁪ Tit

⁪ Hver dag, eller næsten hver dag

1. Hvor ofte i de sidste 3 måneder har du **følt dig usikker** på grund af dine tænder, læber, mund eller kæbe?

⁪ Aldrig

⁪ En eller to gange

⁪ Sommetider

⁪ Tit

⁪ Hver dag, eller næsten hver dag

1. Hvor ofte i de sidste 3 måneder har du **følt dig genert eller forlegen** på grund af dine tænder, læber, mund eller kæbe?

⁪ Aldrig

⁪ En eller to gange

⁪ Sommetider

⁪ Tit

⁪ Hver dag, eller næsten hver dag

1. Hvor ofte i de sidste 3 måneder **har du været bekymret for hvad andre folk tænker om dine tænder, læber, mund eller kæber**?

⁪ Aldrig

⁪ En eller to gange

⁪ Sommetider

⁪ Tit

⁪ Hver dag, eller næsten hver dag

1. Hvor ofte i de sidste 3 måneder har du på grund af dine tænder, læber, mund eller kæbe **været bekymret for, om du ser lige så godt ud som andre**?

⁪ Aldrig

⁪ En eller to gange

⁪ Sommetider

⁪ Tit

⁪ Hver dag, eller næsten hver dag

1. Hvor ofte i de sidste 3 måneder har du **været ked af det (eller irriteret)** på grund af dine tænder, læber, mund eller kæbe?

⁪ Aldrig

⁪ En eller to gange

⁪ Sommetider

⁪ Tit

⁪ Hver dag, eller næsten hver dag

1. Hvor ofte i de sidste 3 måneder har du **følt dig nervøs eller bange** på grund af dine tænder, læber, mund eller kæbe?

⁪ Aldrig

⁪ En eller to gange

⁪ Sommetider

⁪ Tit

⁪ Hver dag, eller næsten hver dag

1. Hvor ofte i de sidste 3 måneder har du på grund af dine tænder, læber, mund eller kæbe **været bekymret for at du ikke er lige så rask som andre**?

⁪ Aldrig

⁪ En eller to gange

⁪ Sommetider

⁪ Tit

⁪ Hver dag, eller næsten hver dag

1. Hvor ofte i de sidste 3 måneder har du på grund af dine tænder, læber, mund eller kæbe **været bekymret for om du er anderledes end andre**?

⁪ Aldrig

⁪ En eller to gange

⁪ Sommetider

⁪ Tit

⁪ Hver dag, eller næsten hver dag

**Spørgsmål om skolen**

1. Hvor ofte i de sidste 3 måneder har du **været væk fra skolen** på grund af smerter eller fordi du skulle til tandlægen.

⁪ Aldrig

⁪ En eller to gange

⁪ Sommetider

⁪ Tit

⁪ Hver dag, eller næsten hver dag

1. Hvor ofte i de sidste 3 måneder har du **haft svært ved at være opmærksom i skolen** på grund af dine tænder læber, mund eller kæbe?

⁪ Aldrig

⁪ En eller to gange

⁪ Sommetider

⁪ Tit

⁪ Hver dag, eller næsten hver dag

1. Hvor ofte i de sidste 3 måneder har du **haft svært ved at lave dine lektier** på grund af dine tænder læber, mund eller kæbe?

⁪ Aldrig

⁪ En eller to gange

⁪ Sommetider

⁪ Tit

⁪ Hver dag, eller næsten hver dag

1. Hvor ofte i de sidste 3 måneder har du **ikke ønsket at tale eller læse højt i klassen** på grund af dine tænder, læber, mund eller kæbe?

⁪ Aldrig

⁪ En eller to gange

⁪ Sommetider

⁪ Tit

⁪ Hver dag, eller næsten hver dag

**Spørgsmål om dine fritidsaktiviteter og samvær med andre**

1. Hvor ofte i de sidste 3 måneder har du **undgået at deltage i aktiviteter som klubber, drama, musik eller skole-udflugter** på grund af dine tænder, læber, mund eller kæbe?

⁪ Aldrig

⁪ En eller to gange

⁪ Sommetider

⁪ Tit

⁪ Hver dag, eller næsten hver dag

1. Hvor ofte i de sidste 3 måneder har du **ikke ønsket at snakke med andre børn** på grund af dine tænder, læber, mund eller kæbe?

⁪ Aldrig

⁪ En eller to gange

⁪ Sommetider

⁪ Tit

⁪ Hver dag, eller næsten hver dag

1. Hvor ofte i de sidste 3 måneder har du på grund af dine tænder, læber, mund eller kæbe **undgået at smile eller le, når du er sammen med andre børn**?

⁪ Aldrig

⁪ En eller to gange

⁪ Sommetider

⁪ Tit

⁪ Hver dag, eller næsten hver dag

1. Hvor ofte i de sidste 3 måneder har du på grund af dine tænder, læber, mund eller kæbe **haft svært ved at spille på musikinstrumenter såsom blokfløjte, klarinet eller trompet**?

⁪ Aldrig

⁪ En eller to gange

⁪ Sommetider

⁪ Tit

⁪ Hver dag, eller næsten hver dag

1. Hvor ofte i de sidste 3 måneder har du **ikke ønsket at være sammen med andre børn** på grund af dine tænder, læber, mund eller kæbe?

⁪ Aldrig

⁪ En eller to gange

⁪ Sommetider

⁪ Tit

⁪ Hver dag, eller næsten hver dag

1. Hvor ofte i de sidste 3 måneder har du **skændtes med andre børn eller din familie** på grund af dine tænder, læber, mund eller kæbe?

⁪ Aldrig

⁪ En eller to gange

⁪ Sommetider

⁪ Tit

⁪ Hver dag, eller næsten hver dag

1. Hvor ofte i de sidste 3 måneder har du **oplevet at andre børn har drillet dig eller givet dig øgenavne** på grund af dine tænder, læber, mund eller kæbe?

⁪ Aldrig

⁪ En eller to gange

⁪ Sommetider

⁪ Tit

⁪ Hver dag, eller næsten hver dag

1. Hvor ofte i de sidste 3 måneder har du **oplevet at andre børn har fået dig til at føle dig udenfor** på grund af dine tænder, læber, mund eller kæbe?

⁪ Aldrig

⁪ En eller to gange

⁪ Sommetider

⁪ Tit

⁪ Hver dag, eller næsten hver dag

1. Hvor ofte i de sidste 3 måneder har du **oplevet at andre børn har stillet dig spørgsmål om dine tænder, læber, kæber eller mund**?

⁪ Aldrig

⁪ En eller to gange

⁪ Sommetider

⁪ Tit

⁪ Hver dag, eller næsten hver dag

**Mange tak for din hjælp!**
